# Development of an automated production process of [^64^Cu][Cu (ATSM)] for positron emission tomography imaging and theranostic applications

**DOI:** 10.1002/jlcr.3973

**Published:** 2022-04-29

**Authors:** Tengzhi Liu, Kathrine Røe Redalen, Morten Karlsen

**Affiliations:** ^1^ Department of Physics Norwegian University of Science and Technology Trondheim Norway; ^2^ Department of Radiology and Nuclear Medicine St. Olavs Hospital, Trondheim University Hospital Trondheim Norway

**Keywords:** ^64^Cu‐ATSM, automated synthesis, hypoxia, PET, radiolabeling, Tracerlab

## Abstract

Cyclotron‐produced copper‐64 radioisotope tracers offer the possibility to perform both diagnostic investigation by positron emission tomography (PET) and radiotherapy by a theranostic approach with bifunctional chelators. The versatile chemical properties of copper add to the importance of this isotope in medicinal investigation. [^64^Cu][Cu (ATSM)] has shown to be a viable candidate for imaging of tumor hypoxia; a critical tumor microenvironment characteristic that typically signifies tumor progression and resistance to chemo‐radiotherapy. Various production and radiosynthesis methods of [^64^Cu][Cu (ATSM)] exist in labs, but usually involved non‐standardized equipment with varying production qualities and may not be easily implemented in wider hospital settings. [^64^Cu][Cu (ATSM)] was synthesized on a modified GE TRACERlab FXN automated synthesis module. End‐of‐synthesis (EOS) molar activity of [^64^Cu][Cu (ATSM)] was 2.2–5.5 Ci/μmol (HPLC), 2.2–2.6 Ci/μmol (ATSM‐titration), and 3.0–4.4 Ci/μmol (ICP‐MS). Radiochemical purity was determined to be >99% based on radio‐HPLC. The final product maintained radiochemical purity after 20 h. We demonstrated a simple and feasible process development and quality control protocols for automated cyclotron production and synthesis of [^64^Cu][Cu (ATSM)] based on commercially distributed standardized synthesis modules suitable for PET imaging and theranostic studies.

## Graphical abstract

Various production methods of [^64^Cu][Cu (ATSM)] exist, but non‐standardized equipment results in varying production qualities often not easily implemented in wider hospital settings. [^64^Cu][Cu (ATSM)] was synthesized on a modified GE TRACERlab FXN automated synthesis module with high molar activity and good radiostability over 20 h. We demonstrate a simple and feasible synthesis of [^64^Cu][Cu (ATSM)] based on commercially distributed standardized synthesis modules suitable for PET imaging and theranostic studies of tumor microenvironment hypoxia.

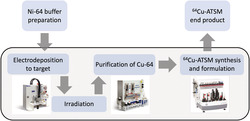

## 1 INTRODUCTION

Positron emission tomography (PET) is one of the most effective modalities for the detection, imaging, and diagnosis of solid tumors. Recently, there has been a growing expansion to use PET in clinics for diagnosis and characterization of solid tumors, not only due to its high sensitivity, but also because of the high degree of flexibility that allows a variety of functional and metabolic assessments of tissue and tumor microenvironments, which in terms provides vital information of tumor properties that are otherwise difficult to obtain.^1^ Tumor hypoxia occurs when rapid tumor proliferation rate outpaces the formation of necessary blood vessels, in combination with the irregularity of tumor vasculature that are typically immature, tortuous, and structurally aberrant.^2^ It has been demonstrated that tumor hypoxia associates with malignant progression and overall poor prognosis and hypoxic tumors are known to be therapeutic resistant, especially for radiotherapy.^3^, ^4^, ^5^

Copper‐64 (Cu‐64) is a synthetic positron emitting radionuclide that can be used for PET.^6^ Compared with other common radionuclides such as fluorine‐18 or carbon‐11, Cu‐64 has the advantage of having a longer half‐life (T_1/2_ = 12.7 h) and a complex decay scheme including β^+^ (17.6%), β^−^ (38.5%), γ (%), and electron capture (43.9%) which generates a cascade of Auger electrons.^7^ Copper is a redox active transition metal that complexes with a variety of ligands, where the affinity between copper and chelators may highly depend on the oxidation state of copper, making it an ideal candidate for oxygenation sensing.^8^ Furthermore, Auger electrons are known to have high linear energy transfer (LET) and are highly cytotoxic.^9^, ^10^, ^11^ Auger electron emission from Cu‐64 provides the possibility of delivering high dose of localized radiation, making it a potential candidate for internal radiation therapy.^12^

Cu‐64 is mainly produced via solid or liquid target ^64^Ni(p,n)^64^Cu reaction in medical cyclotrons. Alternatively, it can also be prepared in lower yields by ^68^Zn(p,αn)^64^Cu reaction.^13^, ^14^ Production of non‐carrier added Cu‐64 using a biomedical cyclotron was first reported by Szelecsényi et al. in 1993 following the ^64^Ni(p,n)^64^Cu reaction.^15^ Later modification of this process have been reported; McCarthy et al. established an efficient production route with high specific activity following the same reaction and has since been widely adapted by other studies.^13^, ^16^, ^17^, ^18^ Among the various methods, the ^64^Ni(p,n)^64^Cu route in a biomedical cyclotron attracted substantial popularity partly due to the simplicity of the reaction and isolation process using ion‐exchange column, and more importantly the increasing availability of biomedical cyclotron facilities in research and hospital facilities, in contrast to the much limited access of a reactor facility. From a general radiotracer production perspective, modern facilities have growing focuses on using automated production modules that can be placed into a fully shielded hot cell for high efficiency, high consistency, and high specific activity productions, in addition to minimizing radiation exposure to the production personnel.^19^ Such synthesis modules have been commercialized and deployed in hospitals and productions sites for the synthesis of PET tracers such as 2‐deoxy‐2‐[^18^F]fluoroglucose ([^18^F]FDG) and 1‐(2‐nitro‐imidazolyl)‐3‐[^18^F]fluoro‐2‐propanol ([^18^F]FMISO).^20^, ^21^, ^22^ On the other hand, these automated synthesis modules also have some associated challenges, including reconfiguration of the synthesis modules between different tracers or usage of multiple synthesis modules for different types of radiopharmaceuticals.^23^ For radiometals, much of the research are still performed using manual or semi‐automated radiosynthesis, often due to a lack of easy and dedicated system for fully automated synthesis. For instance, commercially available synthesis modules dedicated for Cu‐64 production typically produce the raw product copper chloride ([^64^Cu]CuCl_2_) in hydrochloric acid (HCl) as a precursor, followed by manual synthetic chemical processes.^24^ These manual synthetic chemical processes increase radiation exposure to the personnel and environment, especially if an evaporation process is involved (such as for the removal of HCl), and are also prone to human errors which may lead to inconsistency of the final product.

**FIGURE 1:**
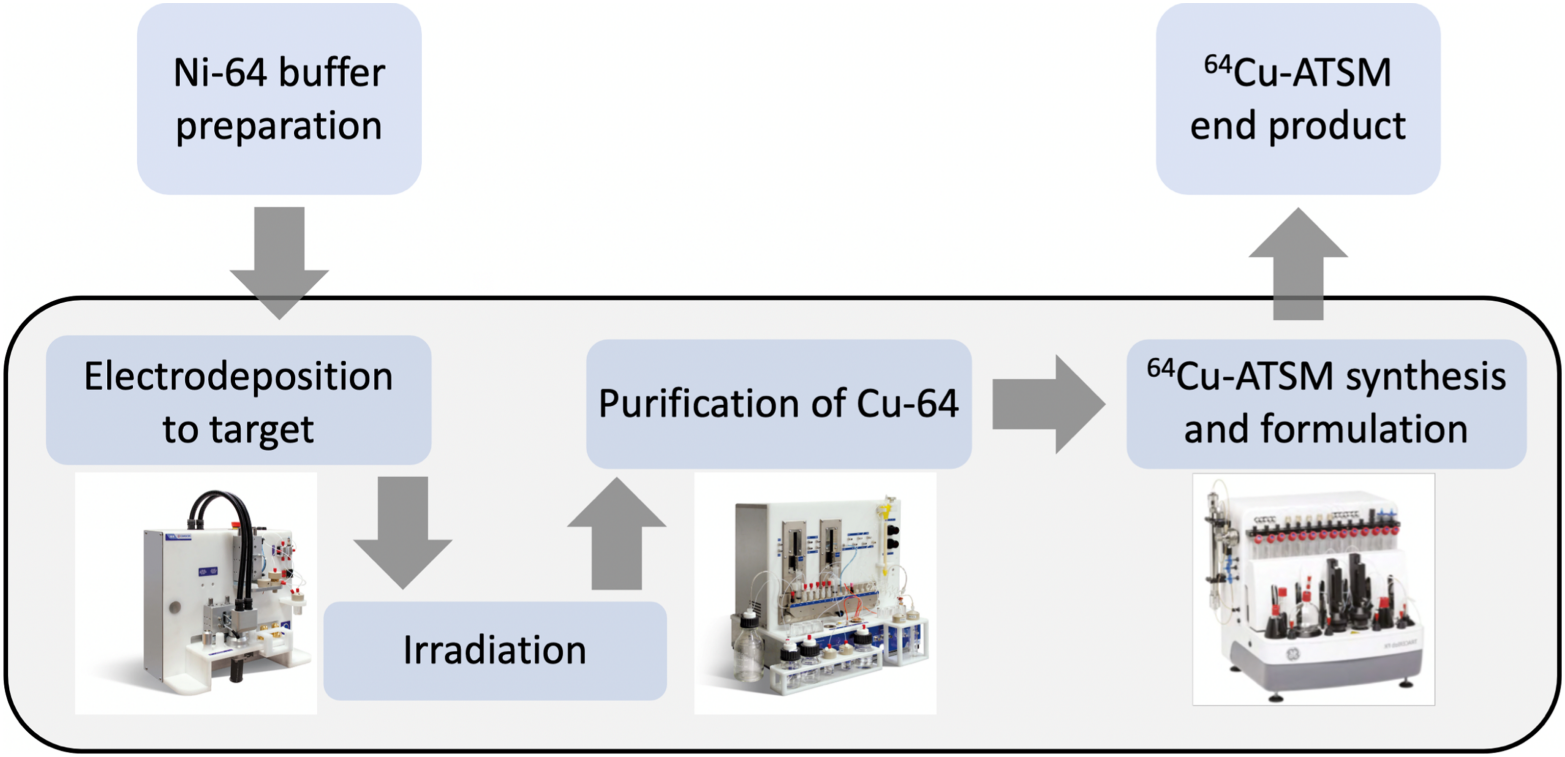
Overview of the synthesis workflow from cyclotron to finished product

Our aim was to develop a flexible and fully automated method for the production of [^64^Cu][Cu (ATSM)], as an example of Cu‐64 radiotracer, based on integration of two commercially available synthesis modules, the *Comecer* “Alceo” system, which involves the dissolution of the irradiated target and purification of Cu‐64 to the raw product [^64^Cu]CuCl_2_, and the *GE* Tracerlab FX2 N synthesis module (Figure 1). The Tracerlab systems have proven to be a versatile, programmable, and efficient automated synthesis module for universal cGMP compliant radiosynthesis of fluorine‐18 and carbon‐11 tracers.^23^, ^25^ Tracerlab FX_FN_ has also been used for the radiosynthesis of generator produced gallium‐68 radiopharmaceuticals with excellent results.^26^, ^27^, ^28^ Recently reported sequential elution approach of ^61/64^Cu^2+^ produced in a liquid target also utilized both cationic and anionic resins with a drying step to remove HCl and further re‐dissolve and elute the copper in buffered solution. This synthesis was performed on an IBA Synthera® Extension synthesizer, which can also be used in the automated synthesis of [^61/64^Cu][Cu (ATSM)] in a cassette‐based synthesis^14^ and also recently demonstrated on a Fastlab (GE Healthcare) cassette‐based system with solid target produced Cu‐61/Cu‐64.^29^

## 2 EXPERIMENTAL

### 2.1 Reagents

All chemicals were obtained from standard commercial sources and used without further purification. Isotopically enriched Nickel‐64 (Ni‐64, isotopic purity >99.52%) used for target preparation was obtained from Isoflex USA (San Francisco, CA). The following reagents were obtained as trace metal purity: concentrated nitric acid (Suprapure®, 65%, Merck Life Science AS, Germany), concentrated hydrochloride acid (Suprapure®, 30%, Merck Life Science AS), ammonium chloride (Merck Life Science AS), ammonium hydroxide solution (28%, Merck Life Science AS), sodium acetate (≥ 99.999%, Merck Life Science AS), Ultrapure water (Honeywell‐Fluka, TraceSELECT for trace analysis), acetonitride (Merck Life Science AS), dimethylsulfoxide (99.7%, extra dry, Acros Organics, Germany), ethanol (absolute, EMSURE® ACS, ISO, Reag. Ph Eur, Merck Life Science AS), sodalime (Merck Life Science AS), and molecular sieves (Acros organics, Germany).

The following reagents were used for the synthesis and purification of the precursor, H_2_‐ATSM: 2,3‐butanedione (97%, Sigma‐Aldrich), 4‐methyl‐3‐thiosemicarbazide (97%, Sigma‐Aldrich), methanol (hypergrade for LC‐MS, Merck Life Science AS), diethyl ether (>99%, Sigma‐Aldrich), and copper (II)‐diacetylbis(*N* ^4^‐methylthiosemicarbazonato) analytical standard for HPLC (>98%, Sigma‐Aldrich).

AG® 1‐X8 anion exchange resin (Bio‐Rad, CA) was used for the purification of [^64^Cu]CuCl_2_. Sep‐Pak Plus C18 cartridges (Waters, MA) were used for the purification of [^64^Cu][Cu (ATSM)]. All solutions were prepared and stored in metal‐free polypropylene centrifuge tubes (VWR International, LLC).

### 2.2 Chemical synthesis of H_2_‐ATSM precursor

The complex ligand precursor diacetyl‐2‐(4‐*N*‐methyl‐3‐thiosemicarbazone)‐3‐(4‐*N*‐amino‐3‐thiosemicarbazone) (H_2_‐ATSM) was synthesized based on a modified version of the method described by Gingras et al.^30^ and Christlieb et al.^31^, ^32^ 4‐methyl‐thiosemicarbazide (1.05 g, 10 mmol) was dissolved in ethanol (30 ml) in a round bottom flask under vigorous stirring. One milliliter of concentrated HCl was added to catalyze the reaction. 2,3‐Butanedione (0.43 g, 5 mmol) dissolved in ethanol (25 ml) was added dropwise into the solution at 50°C. Upon completion of the addition, a light yellow to white precipitate was formed, and the reaction was stirred for additional 2.5 h. The reaction was cooled to room temperature and the crude product was filtered off, washed with ice cold ethanol (2 × 10 ml), diethyl ether (5 ml), re‐dissolved in dried DMSO (10 ml), and placed in a closed environment with diethyl ether at room temperature for vapor diffusion crystallization as described by Holland et al.^33^ The pure H_2_‐ATSM is a white powder crystal after filtering and drying under vacuum (1.2 g, 4.6 mmol). ^1^H, ^13^C NMR, and MS spectra were collected to determine the structure of the product with a 600‐MHz Avance III HD equipped with a 5‐mm cryo probe from Bruker instruments and SampleCase, mass spectrum collected with electro spray ionization in positive mode (ESI‐MS [+]) on a 6470B triple quadrupole. ^1^H NMR (600 MHz, DMSO‐*d*_6_): 10.21 (s, 2H) NH, 8.37 (bq, ^3^J_HH_ = 4.54, 2H) NHCH_3_, 3.02 (d, ^3^J_HH_ = 4.54, 6H) NHCH_3_, 2.21 (s, 6H) CH_3_. ^13^C NMR (150 MHz, DMSO‐*d*_6_): 178.93, 148.44, 31.68, 12.15. ESI‐MS (+): *m*/*z* 260.4.

### 2.3 Electrodeposition of Ni‐64

The electrodeposition follows the Comecer instruction. Briefly, in a metal‐free Falcon tube, 6 ml of trace water was added into 4.6 g of ammonium chloride under vigorous stirring, followed by 8 ml of ammonium hydroxide solution (28%). The pH of the buffer was adjusted by evaporating excess ammonia under mild heat (30°C–35°C). The final pH of the buffer solution was 9.30 ± 0.02 at 25°C.

For the preparation of initial nickel nitrate (Ni (NO_3_)_2_) solution, metallic Ni‐64 (50 mg) was dissolved in 1 ml of 16 M nitric acid (HNO_3_) at 100°C–120°C, until all the metal was dissolved and a green solution was obtained. Upon continue heating, the volume of the solution was reduced to dryness, and 1.0 ml of trace water was added. The Ni (NO_3_)_2_ solution may also be obtained from the recycle of Ni‐64 from the previous production. In such case, the recycle solution containing the nickel chloride (NiCl_2_) was evaporated to dryness, and 5 ml of concentrated HNO_3_ was added until a solution was formed. The solution was once again evaporated at 100°C–120°C until dryness and re‐dissolved in 1 ml of concentrated HNO_3_. The volume of the Ni (NO_3_)_2_ solution was evaporated to dryness, and 1.0 ml of trace water was added for the final Ni (NO_3_)_2_ solution.

The final Ni (NO_3_)_2_ solution (1 ml) was transferred into a metal‐free centrifuge tube. One milliliter of ammonia buffer solution (as previously described) was added into the Ni (NO_3_)_2_ solution, and a deep blue solution was obtained. The solution was then diluted with 2.25 ml of trace water, and the pH was adjusted with ammonium hydroxide solution (28%) to reach pH = 9.30 ± 0.02 at 25°C.

Electrodeposition of Ni‐64 onto the target surface was performed on the Alceo electrodeposition (EDS) module. Ni (NO_3_)_2_ buffer solution was added into a 10 ml vial and circulated to the target surface. Initial voltage was set to ~2.93 V to obtain an initial current of ~45 mA. The electrodeposition was completed after 15 h with constant circulation and voltage, while the current was reduced to ~10 mA. Upon completion, the solution turned to colorless, and a firm layer of Ni‐64 was formed on the platinum target surface.

### 2.4 Radio‐production of [^64^Cu]CuCl_2_

The production of Cu‐64 followed ^64^Ni(p,n)^64^Cu reaction on a GE PETrace 800 series 16 MeV biomedical cyclotron. The initial beam energy was degraded by a foil to 12 MeV to optimize reaction cross section. The target was cooled with helium (at the front) and water (at the back). In a typical experiment, the target is bombarded at 35 μA beam current for 3–6 h and transferred back to the EDS module.

The irradiated Ni‐64 was then dissolved in 6 M HCl at 90 °C and transferred to the Taddeo synthesis module (Comecer, Italy). The solution was loaded on an AG1‐X8 chloride resin (200–400 mesh, BioRad, Hercules, CA, USA). In a three‐step process, unreacted Ni‐64, co‐produced Co‐61 and Cu‐64 product were eluted with 6 M HCl (40 ml), 4 M HCl (20 ml), and 0.1 M HCl (10 ml), respectively. The Cu‐64 fraction was eluted to a shielded vial and transferred to the Tracerlab FX_FN_ (GE Health Care, USA). Total activity of [^64^Cu]CuCl_2_ in the product vial was 4.0–7.2 GBq in 9 ml of approximately 2 M HCl (determined with titration) for a typical 3 h irradiation.

### 2.5 Automated radiosynthesis of ^64^Cu‐diacetyl‐bis(*N* ^4^‐methylthiosemicarbazone) ([^64^Cu][Cu (ATSM)])

The [^64^Cu]CuCl_2_ solution synthesized as previously described was transferred to the reactor of Tracerlab FX2 N synthesis module. The solution was evaporated at 90°C for 30 min under He flow and vacuum to remove excess HCl. The temperature was increased to 130°C to ensure dryness of the [^64^Cu]CuCl_2_. The reactor was then cooled and maintained at 40°C, before sodium acetate buffer (0.5 M, 2 ml, pH = 5.89) was added into the reactor to re‐dissolve the [^64^Cu]CuCl_2_. The precursor H_2_‐ATSM (20 μg in 20 μl DMSO solution) was added into the reactor along with 0.1 ml ethanol, and the reaction mixture was stirred for 10 min.

**FIGURE 2:**
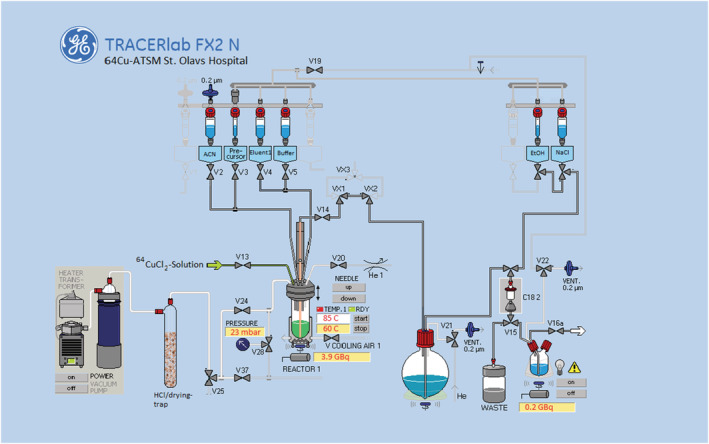
^64^Cu‐ATSM production layout on the Tracerlab FX2 N

The reaction mixture was then transferred to the Sep‐Pak C18 column (preconditioned with 5 ml ethanol and 10 ml water), and the reactor was washed with 3 ml of 20% ethanol in water. The washing was transferred to the column, and eluent was transferred to waste. The [^64^Cu][Cu (ATSM)] product was then eluted with 1 ml of absolute ethanol to dilution flask and diluted with 9 ml of physiological saline solution with 10 mM concentration of sodium ascorbate through the column. The final product was then transferred to a sterile product vial through a Vented Millex GV 0.22 μm sterilization filter. Figure 2 shows the final layout utilized in the Tracerlab FX2 N synthesis module.

### 2.6 Formulation and stabilization of [^64^Cu][Cu (ATSM)]

The formulation of the final [^64^Cu][Cu (ATSM)] was based on a study by Matsumoto et al., as shown in the following table (Table 1).^34^

**TABLE 1:** Typical final formulation of sterile filtered [^64^Cu][Cu (ATSM)] product produced on the module

Content	Quantity
[^64^Cu][Cu (ATSM)]	1 GBq/ml
Ethanol	10%
Physiological saline (0.9%)	4 ml
Sodium ascorbate	10 mM/ml

### 2.7 Radiochemical analysis and quality control

The total radioactivity, activity concentration based on the final volume, and the half‐life of the product were measured in a dose calibrator (CRC‐55tR, Cappintec). Activity yields were calculated using the measured radioactivity before and after the synthesis of radiopharmaceutical.

The radiochemical purity of the radiopharmaceuticals was verified with an Agilent 1260 Infinity II HPLC System equipped with a fraction collector, UV detector (λ = 320 nm for [^64^Cu][Cu (ATSM)], and a radioactivity detector connected in series (Flow‐RAM Radio‐HPLC Detector, Lablogic). Mobile phase composition used for analysis: solvent A (water with 0.1% formic acid) and solvent B (acetonitrile), flow rate was 1.2 ml/min. A reversed‐phase Agilent Poroshell 120 4 μm C18, 100 × 4.6 mm, and an gradient consisting of 80% solvent A and 20% solvent B to 100% during 10 min with total run time of 17 min were utilized for the analysis. The activity of the injected sample was calculated from the volume activity. The molar concentration of [^64^Cu][Cu (ATSM)] was then decay corrected to end of synthesis and calculated from standard calibration curve obtained using reference standard [^nat.^ Cu] [Cu (ATSM)] with the same analysis method prior to the radio synthesis, and the molar activity can be subsequently obtained.

A titration method was created, and a series of titration samples of H_2_‐ATSM was prepared prior to the radiosyntheis.^16^, ^35^ Sodium ascorbate and absolute ethanol were added into the sodium acetate (NaOAc) buffer (0.5 M, pH = 5.89) for stabilization. [^64^Cu]CuCl_2_ (50 μl) obtained from the end of synthesis was added into each titration sample (Table 2). The final concentration of H_2_‐ATSM was 34 nM, 171 nM, 257 nM, 342 nM, 685 nM, 1371 nM, and 2742 nM. The titration samples were then incubated for 10 min and analyzed by radio thin layer chromatography (TLC). A sample of 2 μl was applied on 10 × 3 cm silica on aluminum sheet (Merck) with ethyl acetate as mobile phase. The distribution of radioactivity was determined using a radioTLC Scanner (Lablogic) to determine the ratio of bound versus unbound Cu‐64.

**TABLE 2:** Sample compositions for molar activity titration

Content	Concentration	Quantity
^64^CuCl_2_	0.55 MBq/μl	50 μl
Sodium ascorbate	10 mM	10 μl
Ethanol	100%	100 μl
H_2_‐ATSM	34–2742 nM	400 μl
Total volume		560 μl

Inductively coupled plasma mass spectroscopy (ICP‐MS) is one of the most sensitive method for total quantification of trace metals in liquid samples with good accuracy and resolution. This analysis method was used to quantitatively determine the ionic impurities that can compete for chelator binding in the final product. In the ICP‐MS method, the activity of [^64^Cu][Cu (ATSM)] sample (1 ml) obtained at the end of synthesis was first measured. The sample was stored for 1 week and diluted to 5 ml and then analyzed on ICP‐MS for Cu‐63, Cu‐65, Ni‐64, and zinc‐64 (Zn‐64). Total Cu concentration was determined by adding Cu‐63 and Cu‐65 concentration in the final results. The molar activity of the sample was then determined based on the initial activity at end of synthesis and the concentration of total Cu.

## 3 RESULTS AND DISCUSSIONS

### 3.1 Electrodeposition and irradiation of ^64^Ni target

Electrodeposition overnight yielded an even and firm surface of Ni‐64 on the platinum target. The target has been plated with 40–70 mg of Ni‐64. The plating efficiency is >99.9%, where the remaining Ni‐64 in the electrodeposition buffer is <10 μg/ml. End‐of‐bombardment (EOB) yield was between 0.97 mCi/μA*h (0.80 mCi/μA*h*mg) and 1.47 mCi/μA*h (0.85 mCi/μA*h*mg). In a typical experiment, 50‐mg Ni‐64 bombarded for 3 h at 35 μA yields a total of 4–6 GBq [^64^Cu]CuCl_2_ at the end of synthesis.

**FIGURE 3:**
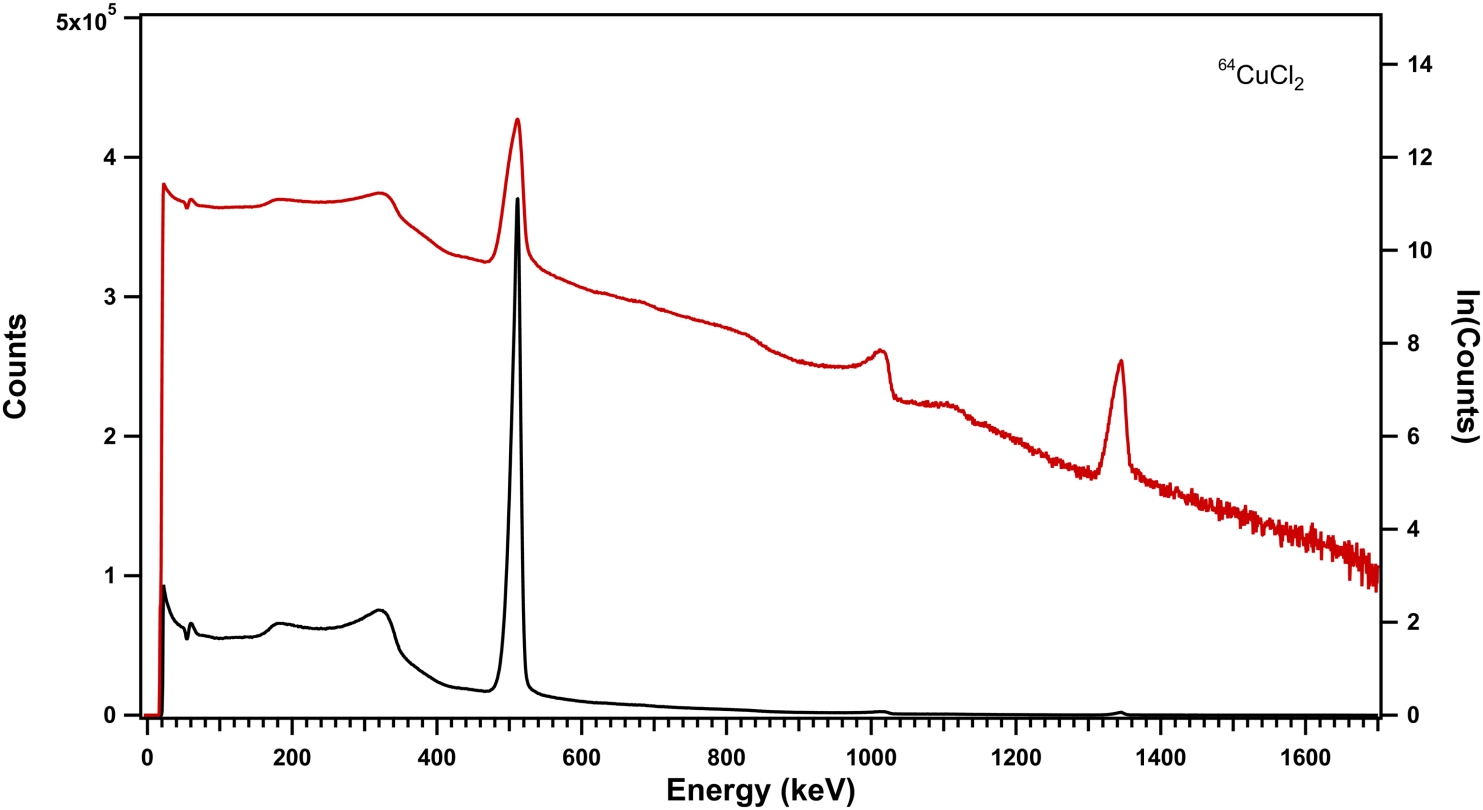
HPGe analysis of the cyclotron produced copper‐64 after purification in the Alceo setup

Radionuclidic purity was determined using a gamma spectrometer (Multichannel Analyzer, HPGe) (Figure 3). The half‐life was measured in a dose calibrator for 6 h to a half‐life of 12.72 h.

### 3.2 Radiochemical synthesis of [^64^Cu][Cu (ATSM)]

#### 3.2.1 Influence of pH

The resulting [^64^Cu][Cu (ATSM)] product from the Alceo module contains approximately 2 M HCl based on titration with 0.1 M sodium hydroxide (NaOH). The eluent for Cu‐64 was adjusted from 0.5 M HCl to 0.1 M HCl, but the HCl in the resulting product remains unchanged mainly due to previous elution of the column with 4 M HCl and the holdup volume of the acid in the column. We found that it is critical in this step that the excess HCl is removed before the chelation with H_2_‐ATSM. Even small amount of the residual acid significantly hampers the chelation efficiency, in which the majority of the Cu‐64 will remain in free ionic form and transferred to waste. The solution was two‐fold; first, acid in [^64^Cu]CuCl_2_ was evaporated under vacuum and He flow at 90°C and 130°C; second, 0.5 M NaOAc buffer was used in the reaction mixture to neutralize any remaining HCl.

One thing noteworthy is that the exhaust vent from the evaporation must be captured/dried by a 1:1 mixture of dried sodalime and activated molecular sieve (200 ml) to prevent corrosion in the TracerLab module and the hotcell. This is illustrated in the schematic of the module as shown in Figure 2. The liquid nitrogen trap after the adsorber collected small amount of water after synthesis and the pH was measured with pH strips and lies in a typical range of pH 4–6 (n repetition > 20). The adsorption trap could be reused several times as long as the adsorbent was thoroughly dried and activated between each cycle (250°C heating in an oven for 20 h).

### 3.3 Molar activity

#### 3.3.1 HPLC, titration, and ICP‐MS

**FIGURE 4:**
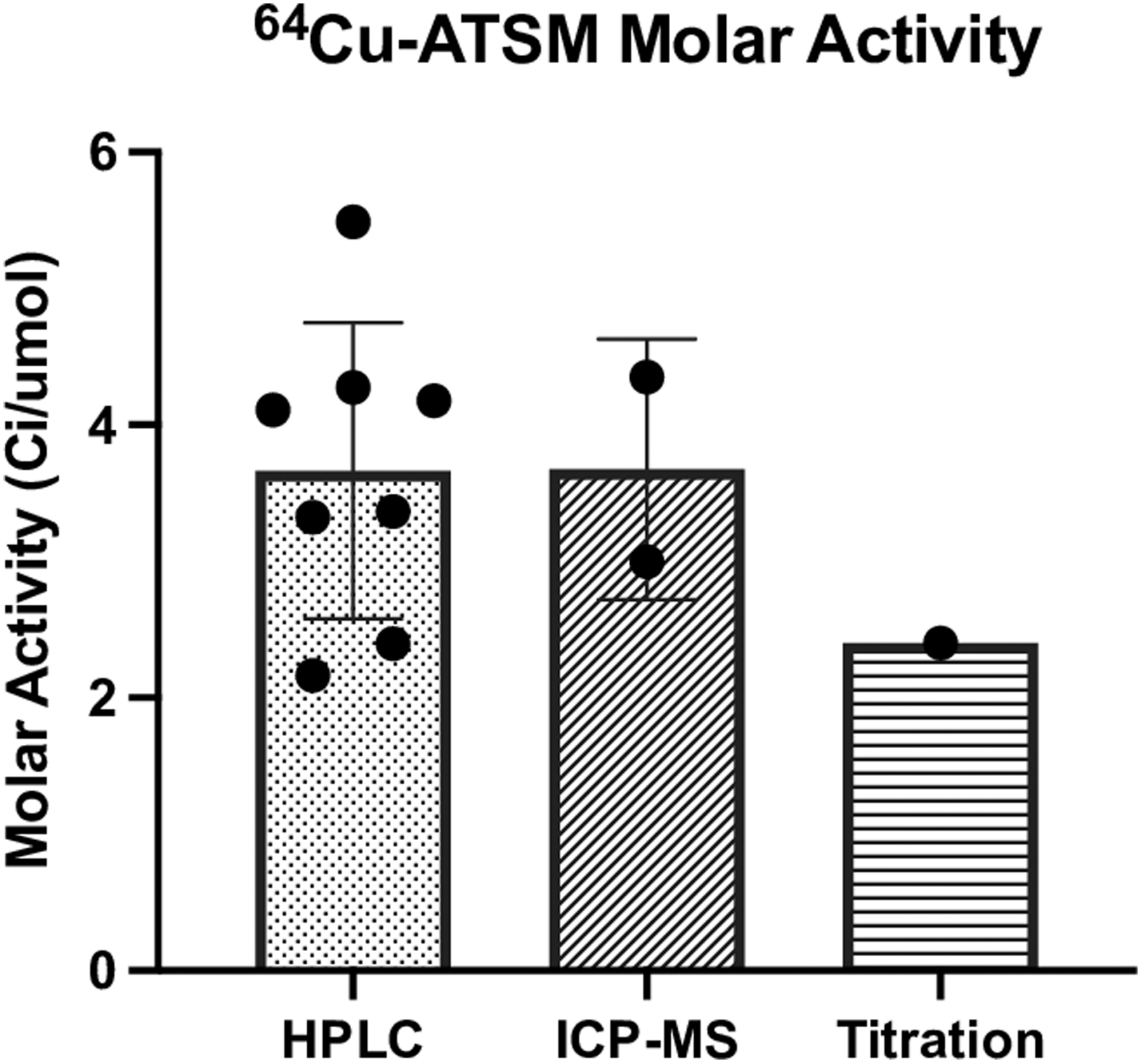
Comparison of the molar activity measured from HPLC, ICP‐MS, and titration

**FIGURE 5:**
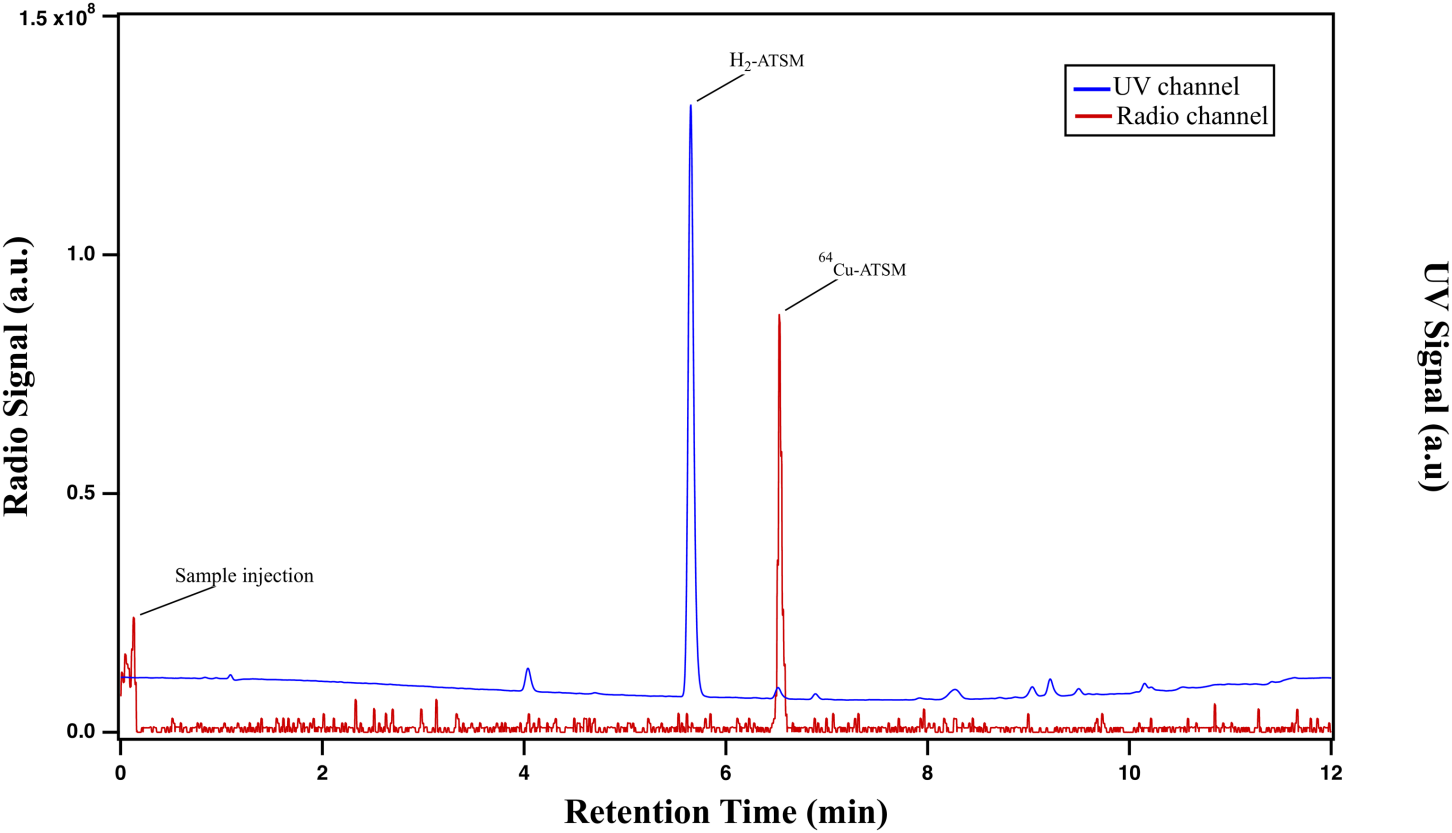
UV signal and radio signal of the finished product on HPLC

It was critical that the molar activity was analyzed and accounted for in the method development of the automated labeling process, to make sure that no extra metal impurities was added from the last labeling step. The molar activity at the end‐of‐synthesis (EOS) of [^64^Cu][Cu (ATSM)] was 2.2–5.5 Ci/μmol (HPLC), 2.3 Ci/μmol (ATSM‐titration), and 3.0–4.4 Ci/μmol (ICP‐MS) (Figure 4) comparable with that of literature values with similar production procedures.^16^, ^36^ Radiochemical purity was determined to be >99% based on radio‐HPLC Traditionally for radiochemical compounds, and the HPLC molar activity is limited by the sensitivity of the UV detection method of the cold compounds. In the case of natural Cu‐ATSM, the limit of detection is relatively low (thus, sensitivity is high), and we were able to quantify the Cu‐ATSM peak using the UV detection at 320 nm. The molar activity reflects the isotopic purity of the compound. The theoretical molar activity of [^64^Cu][Cu (ATSM)] is 9.4 TBq/μmol (254 Ci/μmol),^35^ and one main reason for the measured molar activity to be significantly lower than theoretical value is because of the cold compound contamination. Since the actual Cu‐64 produced is in pmol to nmol region, trace amount of ^nat.^ Cu compares significantly more than the produce Cu‐64. This ratio is dependent upon irradiation time of the target as there are few byproducts in the nuclear reaction. The amount of pure [^64^Cu][Cu (ATSM)] injected into the HPLC system would be below the limit of detection for the UV detector and does not significantly contribute to the detected UV peak, the contribution of the peak is almost purely from the cold compound (Figure 5).

**FIGURE 6:**
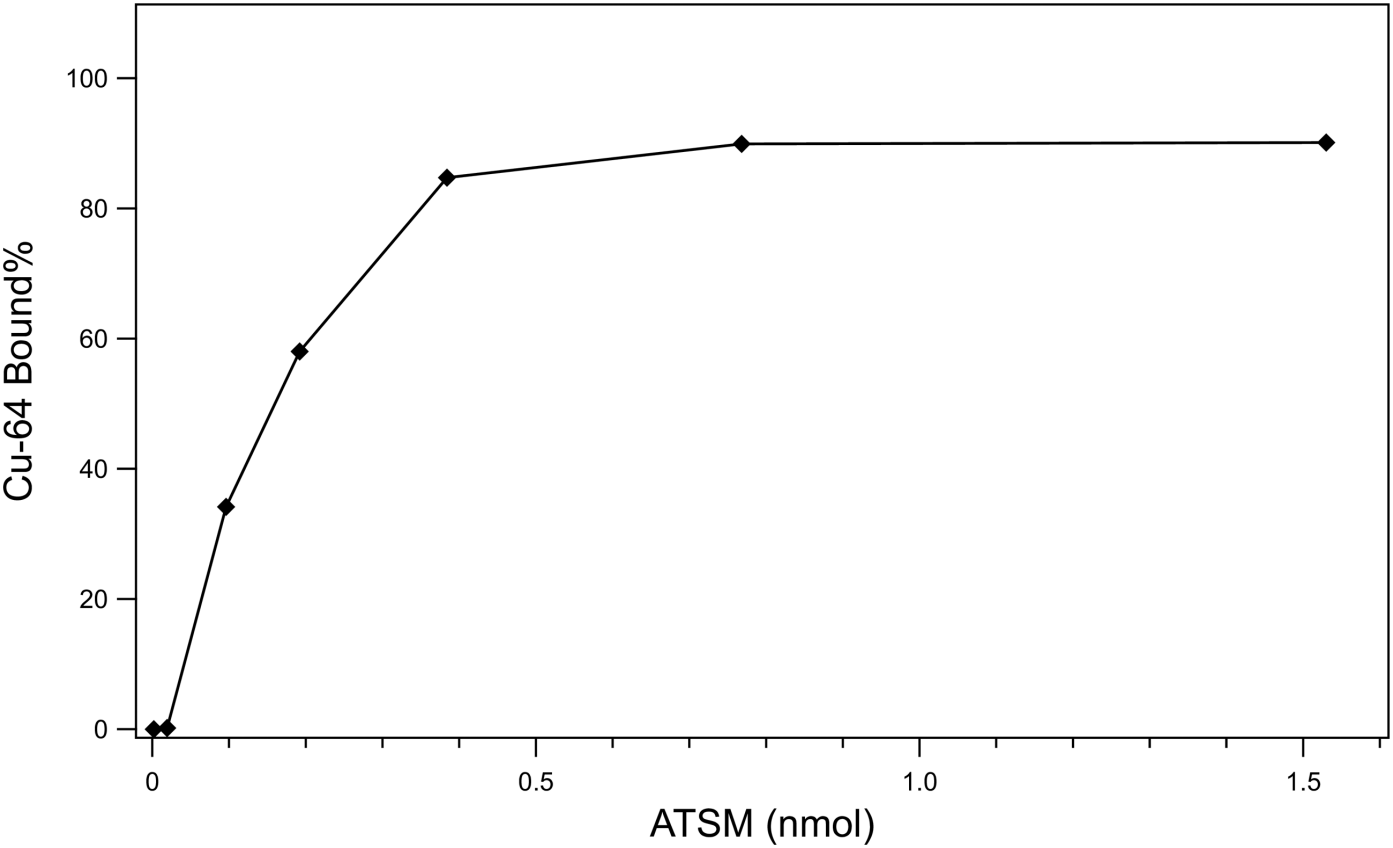
Titration plot showing bound Cu as a function of ATSM chelator amount

In the titration methods, there are two main factors that determines the molar activity: the binding specificity of the chelator, and the binding affinity between the metal and the chelator. This is sometimes referred to as the “apparent molar activity” (AMA). For instance, it has been demonstrated that a common titration compound (2,2′,2″,2‴‐(1,4,8,11‐tetraazacyclotetradecane‐1,4,8,11‐tetrayl)tetraacetic (TETA) often results in low molar activity when comparing to ICP‐MS, which is a result of competing chelation with metal ions other than Cu, thus a lack of binding specificity.^35^ As shown in Figure 6, titration of [^64^Cu]CuCl_2_ with varying concentration of H_2_‐ATSM shown approximately 150 pmol of H_2_‐ATSM in NaOAc buffer (0.5 M, pH = 5.89) resulted in 50% of bound copper at room temperature in 10 min. The molar activity of 100% binding was estimated based on the 50% binding range determined in the titration and analysis by TLC. AMA was calculated from the estimation of which 50% of [^64^Cu] is bounded. Retention values (Rf) from TLC analysis on silica gel plates with ethyl acetate as the mobile phase: R_f_ = 0–0.1 ([^64^Cu]Cu (OAc)_2_ + [^64^Cu]CuCl_2_), R_f_ = 0.5–0.6 ([^64^Cu][Cu (ATSM)] (Figure 6).

**FIGURE 7:**
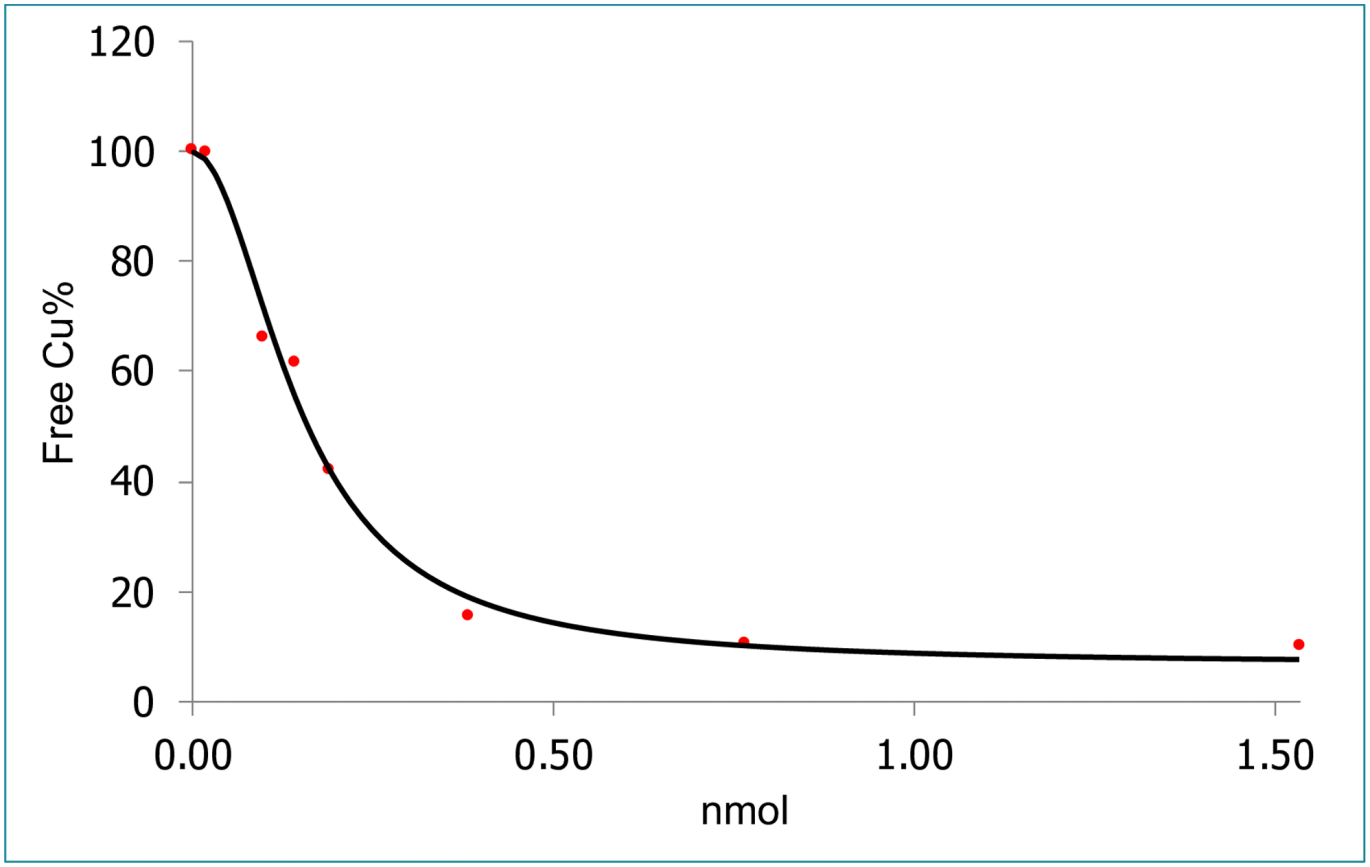
Curve fitting using a 4PL symmetrical sigmoidal plot, X = 0.16 nmol at 50% bound, R^2^ = 0.9892, 

The data were curve fitted using a four‐parameter logistic (4PL) symmetrical sigmoidal plot with X = 0.16 nmol at 50% bound (Figure 7). This resulted in a calculated value (2.3 Ci/umol) comparable with the ones from other methods.

$$Y=\frac{6.481\;+\;\left(100.247\;-\;6.481\right)}{\left(1\;+\;{\left(\frac{X}{0.148}\right)}^{1.912}\right)}$$

Traditionally, the titration was performed using chelators such as TETA or 2,2′,2″‐(1,4,7‐triazacyclononane‐1,4,7‐triyl)triacetic acid (NOTA). Our results show that H_2_‐ATSM can be an equally good, if not better, chelator for the determination of AMA similar to what Obata 2003 describes.^16^ The “true” molar activity (AMA) should preferably be determined with the same chelator as in the final compound composition and in the same chemical and physical environment to determine the selectivity of the radiometal to the chelator from interfering metal ions in the matrix solution used for labeling and in the final formulation. This titration shows that an optimal reaction condition of the labeling of ATSM was achieved in our synthesis method and comparable to the results from the HPLC method.

The ICP‐MS molar activity was calculated based on the total copper found in the decayed samples and the corresponding activity of that same at the end of [^64^Cu][Cu (ATSM)] synthesis. Relevant trace elements including cobalt (Co), nickel (Ni), zinc (Zn), iron (Fe), and lead (Pb) were also measured and compared between the [^64^Cu]CuCl_2_ from the Taddeo module and the end product [^64^Cu][Cu (ATSM)] from the TracerLab synthesis. A summary of results is shown in Table 3.

**TABLE 3:** Results from ICP‐MS analysis of elemental composition for determination of trace metal contribution from the Tracerlab FX2 N

ICP‐MS	Taddeo (μg/L)	Std. dev. (μg/L)	Tracerlab (μg/L)	Std. dev. (μg/L)	Ratio
Fe	21.04	2.2	2.82	0.7	7.46
Co‐59	0.017	15.3	0.017	54.3	1.0
Ni (nat)	0.658	2.9	0.256	1.9	2.57
Cu (63,65)	18.17	1.0	5.5	3.4	3.30
Zn	27.28	0.5	16.28	1.0	1.68
Pb	2.155	2.3	0.058	12.8	37.16

As shown in Table 3, all metal contents tested shown a reduction after the Tracerlab module. The ratio of natural copper content before and after the Tracerlab synthesis is consistent with the radiochemical yield of [^64^Cu][Cu (ATSM)] at the end of the synthesis. The significant reduction of Fe and Pb levels is likely due to the lower binding potential of these metal ions with the ATSM ligand, thus passed through the C‐18 column during the washing step of the synthesis. Ni and Zn are competing metal ions and have similar or better binding efficiency than Cu, thus will be eluted alongside with the product. Noted that Ni‐64 and Zn‐64 are also decayed products of Cu‐64 and that the difficulty in determining Ni‐64 due to the natural abundance of Zn‐64 isobaric overlap will further make the determination of the isotopic composition of these specific isotopes more difficult.

### 3.4 Radiochemical purity and stability

**FIGURE 8:**
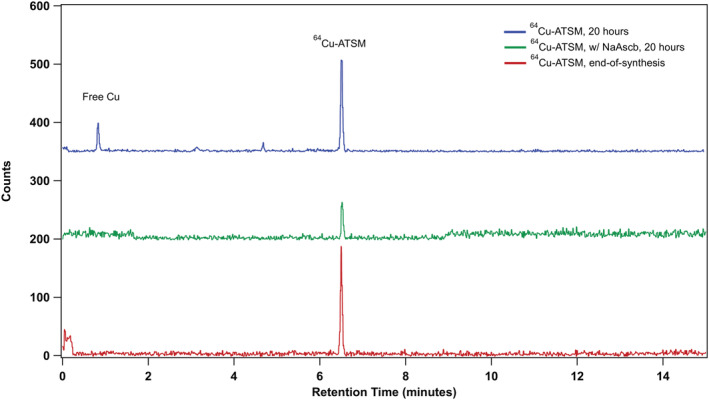
Radio HPLC analysis of the product and the decayed product after 20 h with and without sodium ascorbate formulation.

The radiochemical purity was determined by HPLC with radio detector. The end‐of‐synthesis analysis shows high radiochemical purity (>99%), where almost all the activity was eluted coincides with the retention time of natural Cu‐ATSM standard. Re‐analysis of the samples at various timing showed degradation of [^64^Cu][Cu (ATSM)] due to radiolysis. As shown in Figure 8, after 20 h end‐of‐synthesis, extra peaks from the radio detector are the results of this radiolysis. Here, the radioactive decay (γ, β^+^, β^−^, Auger), specifically the low energy Auger electrons, leads to the accumulation of reactive oxygen species (ROS), which in turn causes decomposition of the [^64^Cu][Cu (ATSM)]. Addition of free radical scavengers, or anti‐oxidant stabilizers such as sodium ascorbate (10 mM/ml), can effectively inhibit the degradation of the final product, which was shown in previous studies as well as demonstrated here (Figure 8) with a similar formulation.^34^

### 3.5 Process development

**FIGURE 9:**
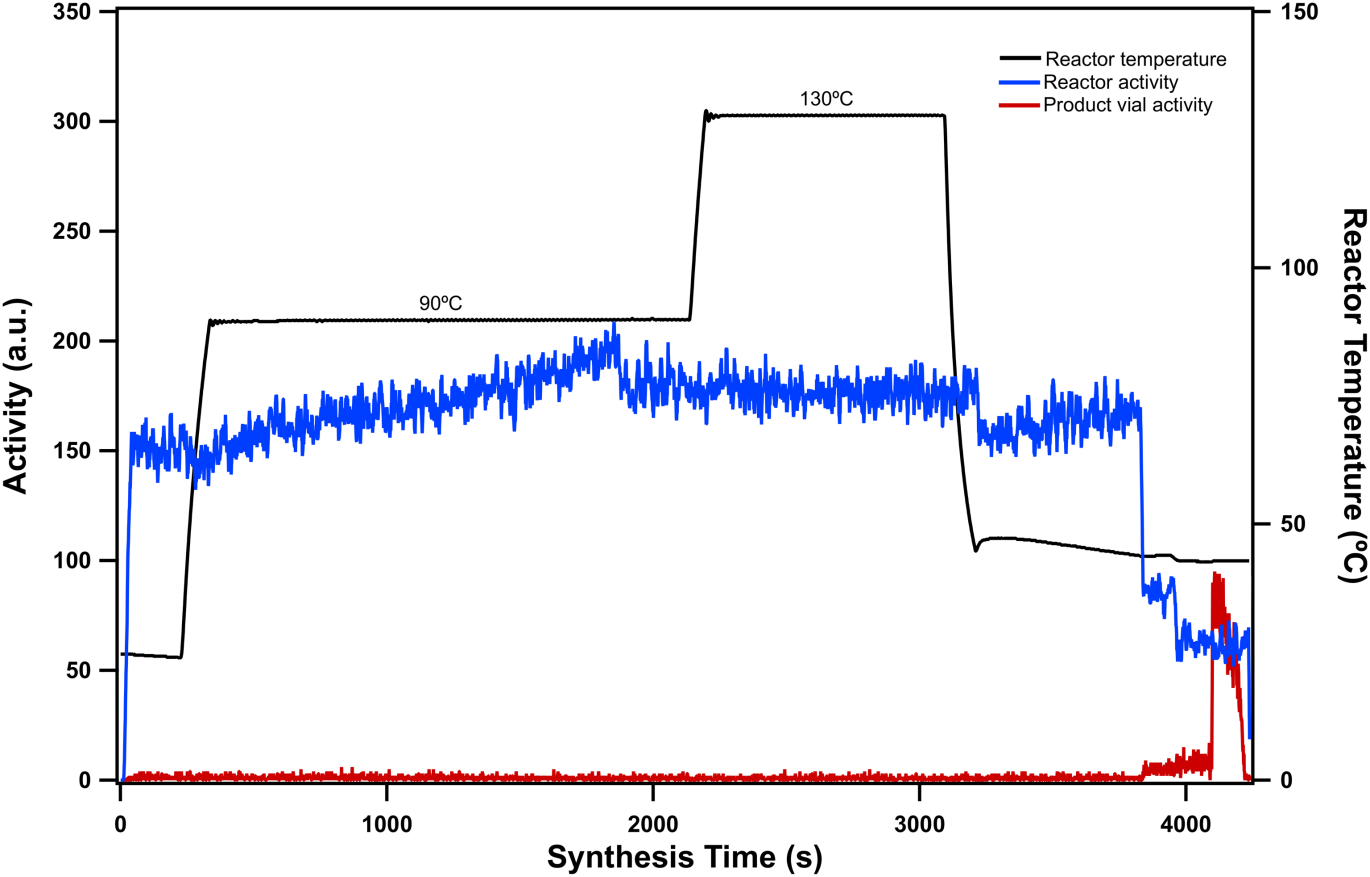
Tracerlab FX2 N report showing the heat profile and activity over the course of the synthesis run

The main focus of this study was to develop an automatic and reliable production and synthesis process for concentration, pH adjustment and labeling of Cu‐64 with ATSM. This process is not only limited to [^64^Cu][Cu (ATSM)] synthesis, but also can be easily adapted to other chelators. The addition of the Tracerlab module enables the possibility to perform traditionally manual steps inside the hotcell automatically. For instance, evaporation of the residual HCl in the [^64^Cu]CuCl_2_ solution can be performed rapidly under controlled heat and vacuum and reconstituted in preferred buffer. This step is significant because the labeling efficiency is highly influenced by the pH; at low pH, protonated chelator cannot effectively bind to the metal ions, which will lead to either low yield or unsuccessful labeling. Conventionally the residual HCl was neutralized by addition of NaOH but will result in an elevated salt concentration, complicating the downstream processes. The Tracerlab module also allows us to automatically add reagents, such as the precursor or solvents, into the build‐in vitreous carbon reactor. In addition, the module allows us to have better control over the final purification and formulation of the radiopharmaceuticals to avoid unnecessary steps where contamination may be introduced. The final temperature profiles and activity levels at different steps can be monitored and reported in final batch documents (Figure 9).

One challenge we encountered is the loss of activity due loss in evaporation tubing and binding of [^64^Cu][Cu (ATSM) to the wall of the reactor. Washing the reactor with ethanol (EtOH) resulted in limited success, while washing with 0.1 M HCl significantly reduced the activity of the reactor. However, in this way [^64^Cu] will be in the form of the free ions. In addition, using excess amount of the precursor H_2_‐ATSM, the loss of [^64^Cu][Cu (ATSM)] can be reduced. Fine tuning of the evaporation time to give copper salt solution in volumes less than 0.5 ml is also possible and well tolerated in the buffer system used. Since the net loss is less than 25% for the overall synthesis, it is considered an acceptable loss for our purpose. Nevertheless, further studies with other solvent or reactor materials may decrease such binding and increase the radiochemical yield. Optimization of the separation method with C‐18 Sep‐Pak column was performed with increasing EtOH concentration for the elution, although the residual H_2_‐ATSM precursor (excess ATSM precursor was added to improve binding efficiency, stability and reaction time) remain mostly unseparated from the [^64^Cu][Cu (ATSM)] without significant loss of activity. This may be improved by using preparative HPLC for the separation, but it will prolong the synthesis time. A restriction loop in front of the C‐18 Sep‐Pak column may be added to have better control over the elution, and thus improve the separation of the product from the precursor.

## 4 CONCLUSIONS

We demonstrate a simple, fast, and versatile process development and quality control protocol for automated production of [^64^Cu][Cu (ATSM)] based on a commercially available synthesis module. End‐of‐synthesis (EOS) molar activity of [^64^Cu][Cu (ATSM)] was 2.2–5.5 Ci/μmol (HPLC), 2.2–2.6 Ci/μmol (ATSM‐titration), and 3.0–4.4 Ci/μmol (ICP‐MS). This result shows good consistency between the three methods used for analysis suggesting high copper selectivity for the chelator (H_2_‐ATSM). Radiochemical purity was determined to be >99% based on radio‐HPLC with good stability over 20 h. The procedure can be easily implemented in hospital PET centers suitable for preclinical PET imaging and theranostic studies. This implementation may also be adapted to studies involving other metal chelators and could increase accessibility of additional copper‐64 based radiopharmaceuticals, in addition to improving consistency of production and radiosynthesis. Further, the fully automated process minimizes personnel radiation exposure.

## Data Availability

The data that support the findings of this study are available from the corresponding author upon reasonable request.
